# Gastrointestinal obstruction due to plaster ingestion: a case-report

**DOI:** 10.1186/1471-2482-6-4

**Published:** 2006-02-16

**Authors:** Rooh- Allah Yegane, Mohammad Bashashati, Reza Bashtar, Mina Ahmadi

**Affiliations:** 1 Department of Surgery, Loqman-Hakim Hospital, Shahid Beheshti University of Medical Sciences, Kamali St, Tehran, Iran; 2 Researcher of Gastroenterology and Surgery, Loqman-Hakim Hospital, Shahid Beheshti University of Medical Sciences Kamali St, Tehran, Iran; 3 General practitioner, Loqman-Hakim Hospital, Shahid Beheshti University of Medical Sciences Kamali St, Tehran, Iran

## Abstract

**Background:**

Plaster ingestion forming gastric bezoar is a strange way to attempt suicide and this method has not yet been reported. It may lead to a mechanical obstruction of the gut, especially the pyloric region, and could manifest with abdominal pain, epigastric distress, nausea, vomiting, and fullness.

**Case presentation:**

Herein we report a case of a 37 year-old woman presenting with plaster ingestion and gastric outlet obstruction, who underwent surgery. At six months follow-up the patient was fully recovered.

**Conclusion:**

Plaster has no toxic or erosive effects. Endoscopic or surgical removing of such material is recommended. Moreover, psychiatric intervention and management is imperative to prevent recurrence in such cases.

## Background

Bezoars are rare causes of gastrointestinal obstruction. They mostly originate in the stomach, and occur mainly in patients with psychiatric ailments who chew and swallow their hair (trichobezoar), vegetable fibres (phytobezoar), persimmon fibres (diospyrobezoar), or tablets/semi liquid masses of drugs (pharmacobezoar) [1-3]. Industrial materials including wood trashes, polystyrene have been reported as the rare causes of bezoar formation [4,5].

In Iran many strange ways are recently used for suicide attempts [6,7]. One of the strangest ways of suicide is plaster ingestion, not have been reported yet.

In this paper, a case of acute formed gastric bezoar due to ingestion of plaster is reported.

## Case presentation

A 37 year old housewife with a previous history of marital conflict was brought to surgical emergency department of Shohadaye-Ashayer Hospital, in Lorestan province situated in the west of Iran. The patient was presented with ingestion of 450 grams of saluted plaster for suicidal attempt. She had eaten the plaster solution seven hours before. The main complaints of the patient were vague epigastric pain, nausea and vomiting.

The Patient had two history of unsuccessful suicidal attempt before. However, beyond all our expectations she had not undergone psychiatric management. She neither smoked nor was an alcoholic, or drug addict.

The patient was hemodynamically stable, but not cooperative. There were no signs of trauma and burning on head and neck. Chest and abdominal physical exams were normal except for a bulging in upper abdomen and a palpable hard mass filling the epigasteric and left upper quadrant area. Laboratory tests were normal.

**Figure 1:**
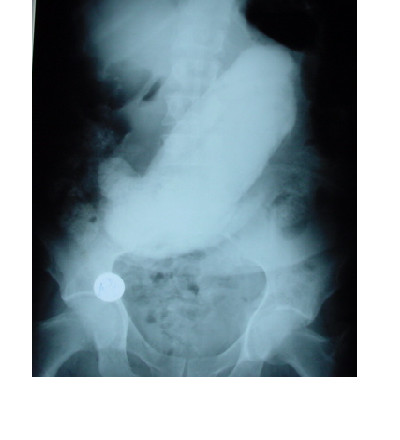
gastric cast on plain abdominal film.

Plain abdominal radiography showed an opaque bezoar as a picture of stomach (Figure-1). According to upper GI flexible endoscopy, esophagus was normal, but endoscope could not enter the stomach. A huge whitish foreign body was seen in stomach. We tried to insert nasogastric tube for irrigation, but it was not successful. The patient was scheduled for emergent surgical exploration.

**Figure 2:**
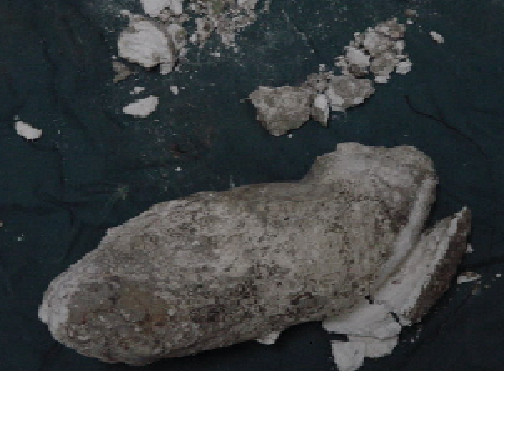
Whitish foreign body removed from the stomach.

Abdomen was opened with upper midline incision. Abdominal exploration was normal. Outer layer of stomach and serosa were normal, but there was a huge hard mass filling the whole cavity of stomach. With longitudinal gastrotomy in greater curvature, stomach was opened. From stomach a round, hard, whitish foreign body was removed (Figure-2). The mucosa was intact. After saline irrigation, stomach was repaired in two layers. Nasogastric tube was inserted.

Liquid diet was started on the 4^th ^day and she was discharged after seven days. Postoperative upper gastrointestinal endoscopic studies did not reveal any inflammation in the stomach. She was referred to the psychiatric consultation and six months of follow-up revealed a satisfactory recovery.

## Conclusion

Most of the reported bezoars are concretions of poorly digested materials. They are usually initiated in the stomach; although some of them may migrate into the bowel or backwardly into esophagus [1,2,4].

Bezoars usually form after a chronic ingestion or consumption of indigestible materials/foods [1,8]. The condition reported here can be named as acute formed bezoar for plaster has been ingested in a moment and formed bezoar in less than 7 hours.

Bezoars may present with abdominal pain, epigastric distress, nausea, vomiting, fullness or bloating[8]. When complicated, diminished peristaltic sounds, rebound, tenderness, distention, diarrhea, constipation, vomiting, and abdominal pain could be found clinically[9]. The main manifestation of our patient was abdominal pain, nausea and vomiting, introducing partial gastric outlet obstruction.

The diagnosis often can be made on the basis of findings of conventional radiography and barium studies [10]. On plain abdominal radiography, we found an opaque bezoar, which formed a perfect cast of the stomach (figure 2). Therefore, the contrast study was not needed.

Endoscopic investigations could show all of gastric bezoars [11]. Bezoars located in the esophagus or stomach should be treated conservatively in the first instance. Surgery is recommended in cases with massive and non-progressive foreign bodies, or complicated cases presenting with perforation, penetration, hemorrhage, or obstruction. Moreover, those causing acute intestinal obstruction require surgical intervention [4,12]. In our patient we tried to fragment the bezoar by endoscope, but we were not successful. Furthermore, our attempt to insert nasogastric tube failed.

Gastric bezoar is commonly removed by longitudinal gastrotomy. If complicated, a few percent of cases can be treated by gastric resections [12]. Our case presented with a large non- movable mass, with manifestations of obstructions. Therefore, gastrotomy and removal of foreign body was performed.

The other part of management of such a bizarre bezoar is thinking whether the ingested material is toxic or corrosive. Plaster or Gypsum (Calcium Sulfate Dihydrate) is a non-toxic agent, which can release nuisance dust in handling or during use. In this manner it may affect eye, skin, nose, throat and upper respiratory tract. Prolonged and repeated exposure can result in lung disease (i.e., silicosis) and/or lung cancer. Incidental ingestion of a sufficient quantity could lead to a mechanical obstruction of the gut, especially the pyloric region[13]. Also, we did not detect any systemic and localized toxicity in our patient. Therefore, no specific care was needed for intoxication.

As most of the patients with bezoar were suffering from psychiatric disorders, psychiatric intervention and management is found to be imperative to prevent recurrence in such cases. More importantly, suicidal attempt of our patient made this part of management more necessary.

## Competing interests

The author(s) declare that they have no competing interests.

## Authors' contributions

All four authors contributed equally in writing of this report.

## Pre-publication history

The pre-publication history for this paper can be accessed here:
